# Band structure tuning of g-C_3_N_4_ via sulfur doping for broadband near-infrared ultrafast photonic applications

**DOI:** 10.1515/nanoph-2021-0549

**Published:** 2021-11-17

**Authors:** Li Dong, Hongwei Chu, Shiping Xu, Ying Li, Shengzhi Zhao, Dechun Li

**Affiliations:** School of Information Science and Engineering, Shandong University, Qingdao 266237, China; School of Environmental Science and Engineering, Shandong University, Qingdao 266237, China; Key Laboratory of Colloid and Interface Chemistry, Ministry of Education, and School of Chemistry and Chemical Engineering, Shandong University, Jinan 250100, China

**Keywords:** broadband, first-principles calculation, optical nonlinearity, sulfur-doping, ultrafast modulation

## Abstract

Graphitic carbon nitride (g-C_3_N_4_) featuring a stable heptazine ring structure and high polymerization degree, was indexed as a high thermochemical stability material, attracting rising research enthusiasm for diverse applications. However, the poor near-infrared (NIR) optical absorption and resulting limited NIR applications were pronounced for g-C_3_N_4_ due to its large bandgap of 2.7 eV. In the present work, sulfur-doping was manifested by first-principles calculations to introduce impurity level and result in anisotropic spin splitting in g-C_3_N_4_ for enhancing broadband nonlinear optical characteristics in NIR regime. The modified sulfur-doped g-C_3_N_4_ (S-C_3_N_4_) exhibited the maximum effective nonlinear absorption coefficient to be −0.82 cm/GW. Pulse duration within hundred nanoseconds was realized with high modulation stability employing S-C_3_N_4_ as saturable absorber in Q-switching operations. Moreover, broadband ultrafast photonics properties were successfully demonstrated in constructed ytterbium-doped and erbium-doped fiber lasers, generating highly stable dissipative soliton and traditional soliton mode-locking pulses. The presented S-C_3_N_4_ nanomaterial with remarkable nonlinear optical performances might explicitly boost the development and application of g-C_3_N_4_ materials in advanced optoelectronic and ultrafast photonic devices.

## 1 Introduction

Nonlinear optical (NLO) materials, as a flourishing fundamental constructing block of advanced laser optics, optoelectronics and optical communication, have set off a huge research boom [1], [2], [3], [4]. Due to their idiographic mechanical, optical and electronic structure virtues absent in the bulk counterparts, the nanomaterials excellently satisfied the growing demands of broadband response, device compactness and high efficiency in ultrafast photonic and optoelectronic fields [5], [6], [7], [8]. Among, carbon nitride (C_3_N_4_), as a metal-free conjugated polymer connected by the *sp* ^2^ hybridization of carbon and nitrogen, has constructed a new class of multifunctional nanoplatforms for electronic, photocatalytic, nonlinear optic, and energy storage applications [9, 10]. There are several allotropes of C_3_N_4_ with distinct structure properties, such as *α*-C_3_N_4_, *β*-C_3_N_4_, cubic C_3_N_4_, pseudocubic-C_3_N_4,_ and graphitic C_3_N_4_ (g-C_3_N_4_) [10]. g-C_3_N_4_ exhibits the most extraordinary thermal and photochemical stability up to 600 °C benefiting from its heptazine ring structure and high degree of polymerization [11]. Therefore, it has been widely applied in photocurrent, photoreactivity, photoelectrocatalysis, and bioimaging domains [11], [12], [13]. Moreover, in terms of its optical properties, g-C_3_N_4_ has been manifested to feature nonlinear saturable absorption and optical limiting properties in the visible waveband [14]. Currently, it has been also integrated into the laser resonator as a saturable absorber (SA), achieving passive Q-switching, and mode-locking infrared pulses [15], [16], [17]. Nevertheless, the large bandgap around 2.7 eV of g-C_3_N_4_ makes its absorption peak near 459 nm, resulting in the poor near-infrared laser absorption [11]. Besides, the relatively small specific surface area, low electron–hole pair separation efficiency, and poor charge mobility of g-C_3_N_4_ restrict its practical optical application as well [18].

To get rid of these drawbacks, various modification strategies have been attempted, such as elemental doping [19, 20], carbon decoration [21], heterojunction formation [22], mesoporous morphology modulation [23], and structure optimization [24] of g-C_3_N_4_. Doping modification, especially anion doping, has been manifested to be a valid scheme to modulate the bandgap, efficiently improving the mobility of photo-induced charge carriers and photoreactivity [25, 26]. Specially, the nonmetallic anion doping could perfectly preserve the metal-free polymerization system [27]. In accordance with the first-principle calculations for elements doping, sulfur was the most chosen dopant as its similar electronegativity and comparable radius with nitrogen [28]. Moreover, massive studies have demonstrated that sulfur doping could distinctly improve the optical and electronic characteristics of g-C_3_N_4_ by narrowing down the bandgap structure, improving near-infrared (NIR) laser absorbance, and accelerating charges separation and carrier mobility [21, 29, 30]. However, the effect of sulfur-introduced defects on broadband third-order nonlinear optical characteristics was rarely reported and lacked internal physical mechanism analysis. Even the research on the performance of sulfur-doped g-C_3_N_4_ in terms of ultrafast modulation characteristics is blank.

Herein, sulfur-doped g-C_3_N_4_ nanomaterials were fabricated by the sonication assisted liquid phase exfoliation method. The porous surface morphology and effectiveness of sulfur doping were characterized by several optical instruments. Subsequently, the broadband nonlinear optical (NLO) absorption properties of the as-synthesized S-C_3_N_4_ were investigated by open-aperture (OA) Z-scan technique. Intensity dependent nonlinear absorption coefficients and modulation depth were observed with a maximum effective nonlinear absorption coefficient to be −0.82 cm/GW. Besides, employing the close-aperture (CA) Z-scan experiments determined its large nonlinear refractive index and third-order nonlinear susceptibility. In virtue of the strong NLO responses in the NIR regime, the as-prepared S-C_3_N_4_ nanomaterials were incorporated into passively Q-switched (PQS) lasers cavities as the SA. The shortest Q-switching pulse width of 87 ns was generated at 1064 nm. The broadband ultrafast mode-locking properties of the S-C_3_N_4_ samples were manifested in ytterbium-doped fiber (YDF) and erbium-doped fiber (EDF) lasers with a high signal-to-noise (SNR) up to 70 dB. What’s more, the density functional theory calculation demonstrated that sulfur-doping introduced defects level and caused anisotropic spin splitting in g-C_3_N_4_ beneficial to the nonlinear optical absorption characteristics of S-C_3_N_4_ in NIR regime. Therefore, the experimental and theoretical results indicated that sulfur-doped g-C_3_N_4_ featured tremendous potential in ultrafast photonic fields and an indelible positive impetus for the development of NIR nonlinear photonic devices.

## 2 Fabrication and characterization

g-C_3_N_4_ nanomaterials were fabricated by the hydrothermal method and liquid-phase exfoliation method. First, 1.773 g thiocyanuric acid (TTCA) and 1.2612 g melamine (MT) was dissolved into 200 and 125 mL hot water (100 °C), respectively. After that, these two dispersions were blended and subjected to magnetic stirring for 30 min. Then it was transferred into an autoclave for 4 h keeping the temperature of 100 °C. After cooling naturally to room temperature, the precipitates were collected and washed by ethanol for several times. Subsequently, the mixture was dehydrated in a vacuum oven for 24 h at 80 °C. The final sulfur-doped g-C_3_N_4_ product was then obtained by calcining the dried mixture in furnace at 550 °C for 4 h. The collected sulfur-doped g-C_3_N_4_ precipitates were dispersed into deionized water and followed by bath ultrasonication for 3 h to disperse the samples thoroughly. Afterward, the resultant dispersion was centrifuged for 30 min at a speed of 12,000 rpm. Then the supernatant containing sulfur-doped g-C_3_N_4_ samples was added dropwise and spun onto the quartz substrate cleaned by ultrasonic treatment in advance. The substrate with attached sulfur-doped g-C_3_N_4_ samples was rotated at a low speed of 1000 rpm in order to disperse the sample uniformly. Finally, the sample was put in a vacuum oven with a constant temperature of 60 °C for 24 h. Thereupon, the uniform dispersed sulfur-doped g-C_3_N_4_ nanomaterials were successfully fabricated for the following experiments.

**Figure 1:**
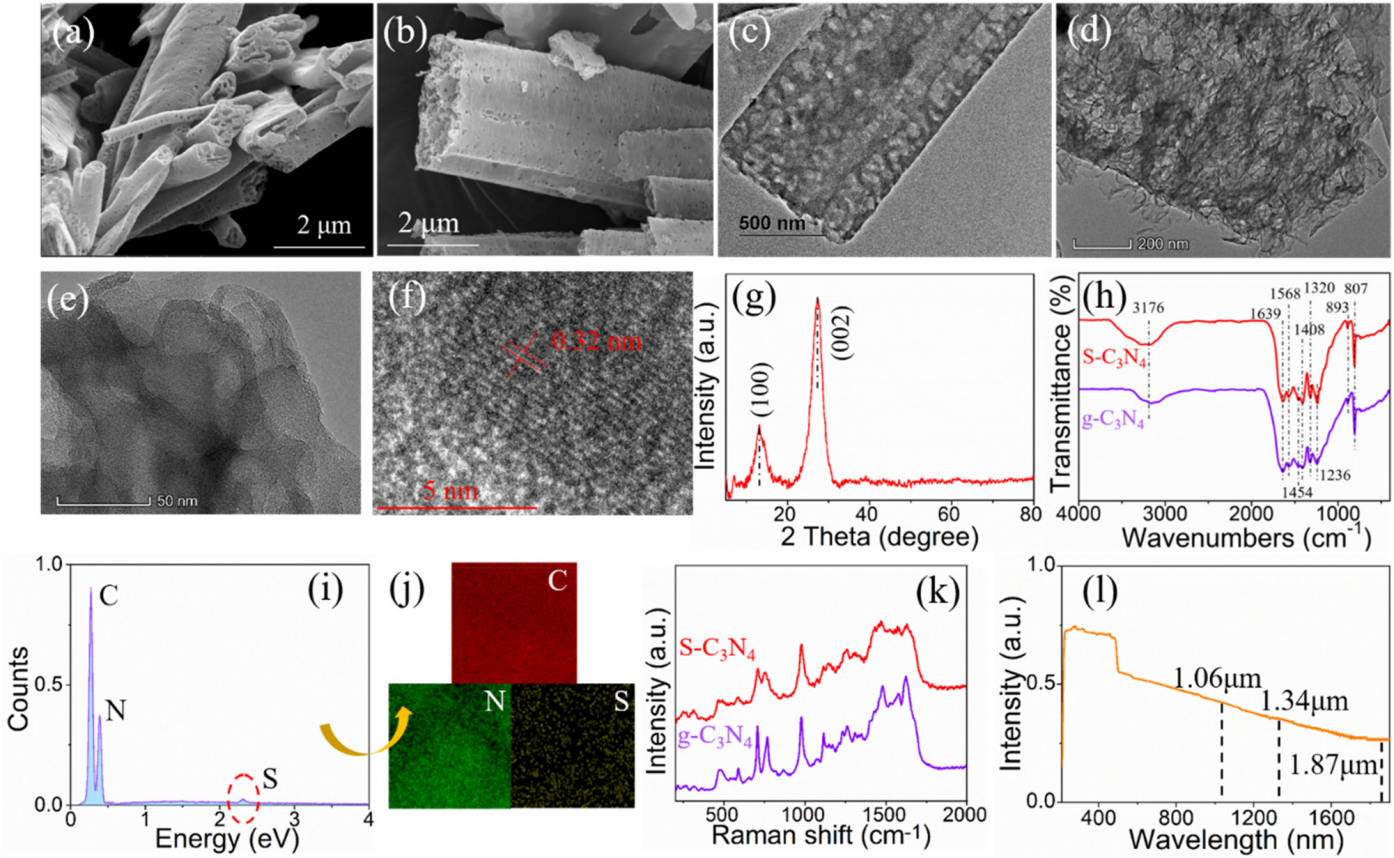
Morphology characterization of the as-prepared S-C_3_N_4_: (a and b) SEM images. (c–f) TEM images with different scales. (g) XRD spectrum. (h) FTIR spectra. (i) EDS image. (j) Elements distribution. (k) Raman spectra. (l) UV–Vis-NIR spectra.

The morphology and microstructure of the S-C_3_N_4_ were characterized via scanning electron microscope (SEM) and transmission electron microscope (TEM). As shown in Figure 1a, the prepared S-C_3_N_4_ powder exhibited a bar-like shape and the front showed abundant honeycomb structures. What’s more, Figure 1b depicted the surface nanoporous morphology distinctly, which could be attributed to the decomposition of thiocyanuric acid during the calcination process at the elevated temperature [31]. The formation of the massive pores and honeycomb structures would exceedingly increase the specific surface area and might bring more surface defects affecting the absorption properties of S-C_3_N_4_. The TEM images further identified the pores structures and Figure 1e showed the layered structures of the as-prepared S-C_3_N_4_ nanomaterials. As shown in the high resolution TEM (HRTEM) image (Figure 1f), a lattice plane separation of 0.32 nm, corresponding to the interlayer distance, indexed to the (002) crystallographic plane of g-C_3_N_4_ [32]. The crystal structure and lattice constant information of S-C_3_N_4_ was determined precisely by X-ray diffraction (XRD) between 5° and 80°. As illustrated in Figure 1g, the diffraction peak located at 13.1°, was ascribed from (100) plane by JCPDS: 87–1526 of graphitic materials, revealing the in-plane structural packing motif, while the representative interlayer stacking (002) peak examined at 27.2°, demonstrated the typical graphite-like stacking of the conjugated aromatic C–N segments [33], consisting well with the XRD results. In addition, sulfur-doping caused no obvious diffraction peak shift and shrink, indicating the well-preserved crystalline structure g-C_3_N_4_. Meanwhile, Fourier transform infrared (FTIR) spectra of the pristine g-C_3_N_4_ and the S-C_3_N_4_ were depicted in Figure 1h, in which the presented characteristic peaks at around 1639, 1568, 1454, 1408, 1320 and 1236 cm^−1^ could be attributed to the stretching vibration modes of the aromatic C–N and C=N heterocycles [34]. Moreover, the peaks located at 807 cm^−1^ reflected the breathing mode of the heptazine ring system, revealing the intact-preserved graphitic C–N network, while the peaks at 3176 cm^−1^ could be ascribed to the terminal NH_2_ or NH stretching vibration [35]. Comparing the FTIR spectra of S-C_3_N_4_ and g-C_3_N_4_ sample, these two transmission spectra showed a slight difference around 893 and 1320 cm^−1^, but owing to the less sulfur dopant in the S-C_3_N_4_ sample, no obvious sulfur peak was found in the FTIR spectrum of the S-C_3_N_4_ sample. The element distribution was detected by an energy dispersive spectrometer (EDS), as shown in Figure 1i and j. Obviously, the sulfur element was uniformly distributed in the as-prepared S-C_3_N_4_ nanosheets, revealing the effectiveness of sulfur doping. The Raman spectrum was employed to illustrate the atom vibrations modes and chemical structure of the g-C_3_N_4_ before and after S-doping. As shown in Figure 1k, several major characteristic peaks were presented at 476, 705, 765, 978, 1479 and 1622 cm^−1^ for pure g-C_3_N_4_ sample. The 476, 705 and 765 cm^−1^ peaks were related to the in-plane symmetrical stretching and the twisting vibration of heptazine CN heterocycles existing in the g-C_3_N_4_ network [36]. The additional peaks were ascribed to the stretching modes of the C–N and C=N bonds. By comparing it with the Raman spectra of S-C_3_N_4_, almost all peaks were observed on S-C_3_N_4_, which demonstrated the well-preserved atomic structure after sulfur doping. However, the intensities of most characteristic peaks were significantly decreased after sulfur doping, which might be due to the fact that sulfur replaced some nitrogen, causing a small amount of damage to the CN heptazine ring systems. What’s more, to examine the broad absorption property of S-C_3_N_4_, the UV-Vis-NIR spectra was characterized, as shown in Figure 1l. Its exhibited obvious broadband absorption in 1∼2 μm waveband laid a solid foundation for the following NIR ultrafast photonic applications.

**Figure 2:**
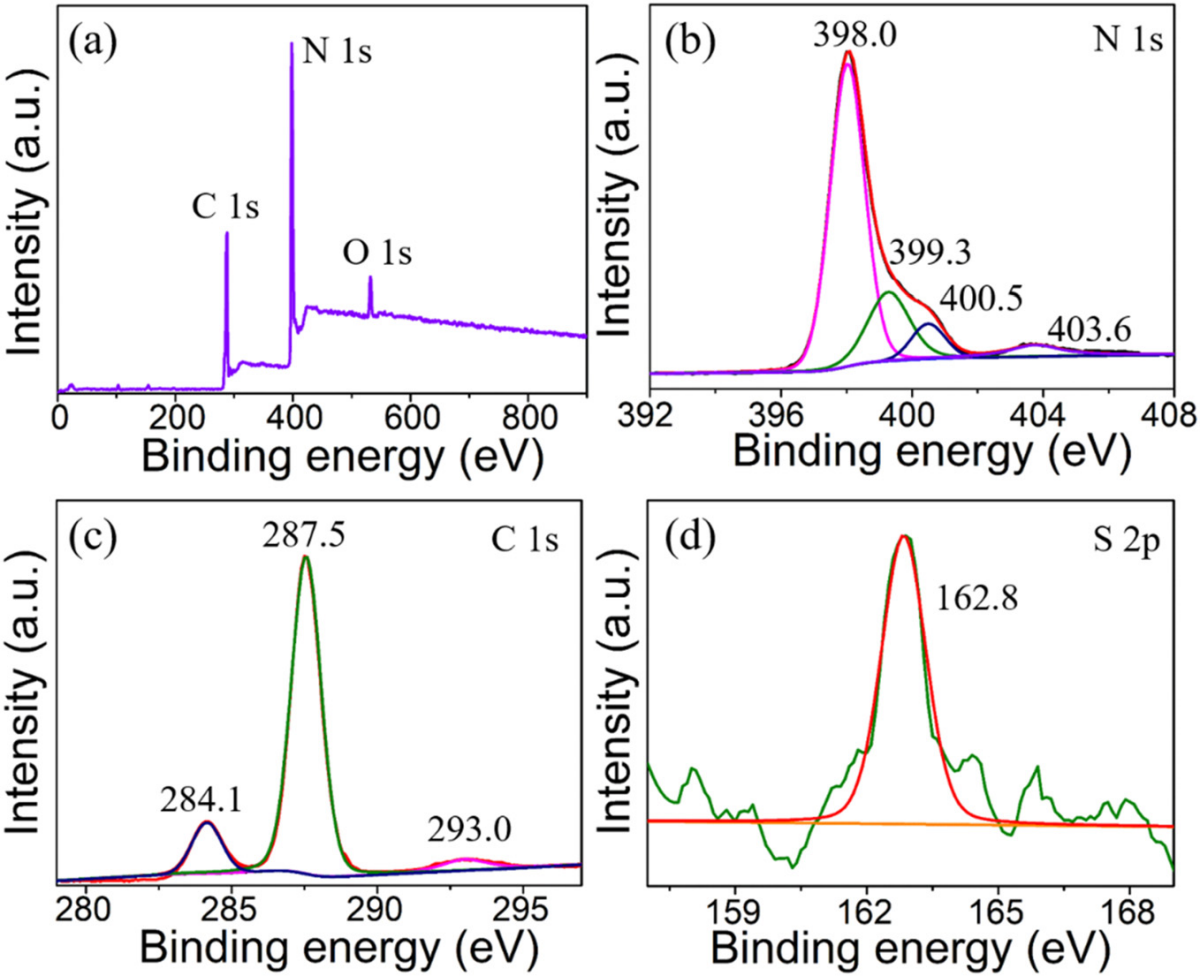
(a) XPS spectrum of the as-prepared S-C_3_N_4_. High-resolution XPS spectrum of (b) N 1*s*, (c) C 1S, and (d) S 2*p* of S-C_3_N_4_, respectively.

To further evidence the effectiveness of sulfur doping and chemical state of the elements of S-C_3_N_4_, the X-ray photoelectron spectroscopy (XPS) technique was performed. As depicted in Figure 2a, carbon, nitrogen and oxygen (caused by the absorbed H_2_O or CO_2_) characteristic peaks were exhibited distinctly, whereas the characteristic peak for sulfur was less prominent due to the low dopant. Therefore, the higher resolution spectra of these elements were investigated to obtain the detailed information of their surface chemical states. First, high-resolution spectra of N 1*s* displayed four characteristic peaks at 398.0, 399.3, 400.5, and 403.6 eV, which could be ascribed to a *sp* ^2^-hybridized nitrogen atom bonded to a carbon atom (C=N–C), tertiary nitrogen N–(C3), nitrogen in the terminal amino group (N–H), and *π*-excitations in g-C_3_N_4_, respectively [37, 38]. Additionally, the C 1*s* spectra could be fitted into three peaks at 284.1, 287.5, and 293.0 eV, as shown in Figure 2c. The main peak at 287.5 eV was attribute to a *sp* ^2^-hybridized carbon in an N-containing aromatic ring (N–C=N) in the g-C_3_N_4_ lattice. The peaks at 284.1 and 293.0 eV was assigned to the *sp* ^2^ C–C bonds and carbon connected to the terminal amino group of g-C_3_N_4_, respectively [39, 40]. Importantly, in the S 2*p* region, a peak around 162.8 eV could be fitted and it was ascribed to C–S bond, demonstrating the replacement of lattice nitrogen with sulfur in S-C_3_N_4_ [41]. Thus, the characterization results demonstrated the successful synthesis of S-C_3_N_4_.

## 3 Experimental results and discussion

### 3.1 Broadband NLO responses

To examine the broadband NLO absorption properties of the modified S-C_3_N_4_ nanomaterials, the incident intensity dependent OA Z-scan experiments were employed at 1064, 1340 and 1878 nm, respectively. The detailed description of the experimental details was attached in the Supplementary Material. In addition, the CA Z-scan technique was implemented to investigate the nonlinear index and the third-order susceptibility. Subsequently, the prepared S-C_3_N_4_ SA was employed in the bulk and fiber lasers to realize the Q-switching and mode-locking operation.

**Figure 3:**
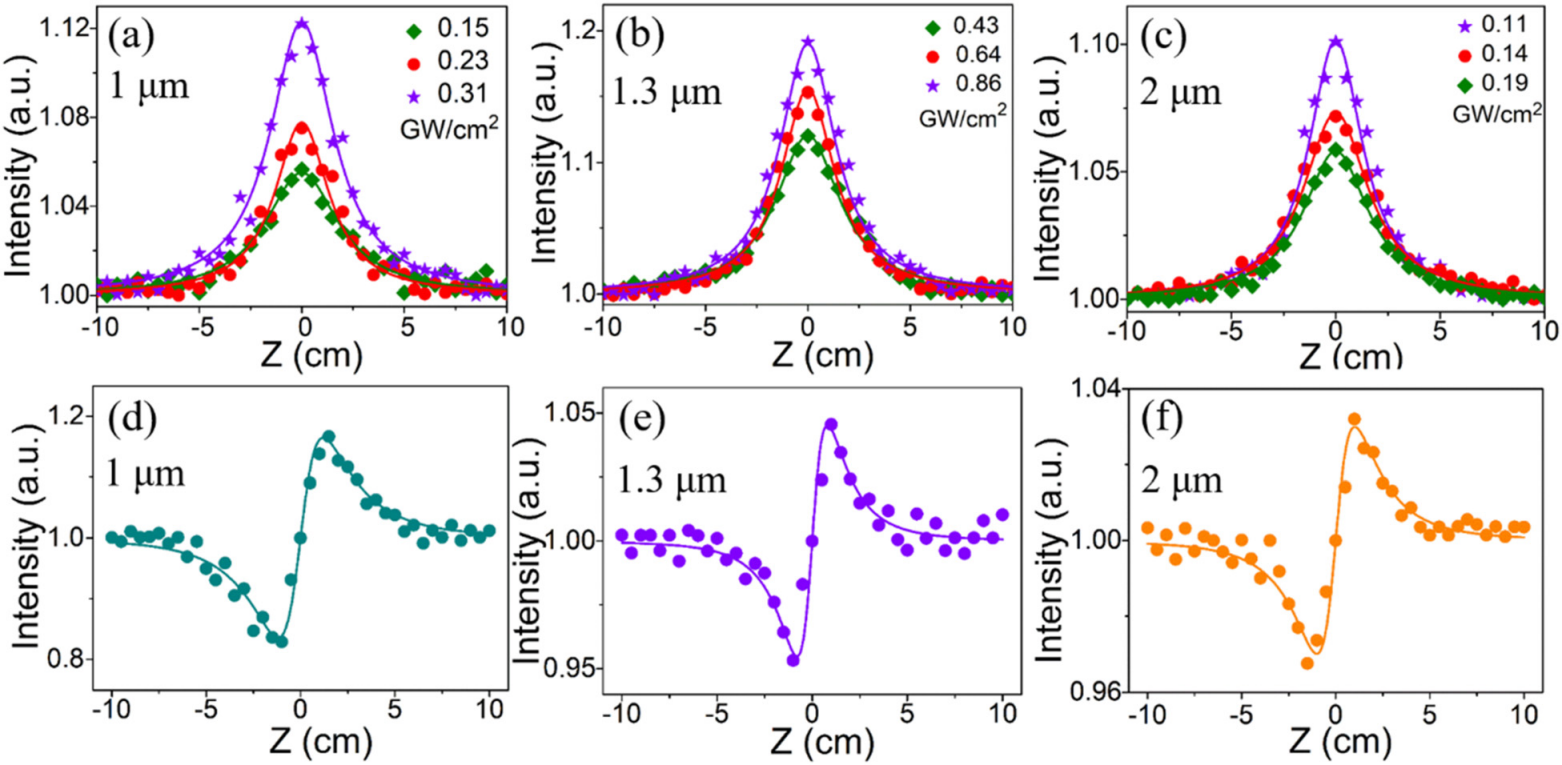
OA Z-scan results of S-C_3_N_4_ at (a) 1.06, (b) 1.34, and (c) 1.87 μm, respectively. CA Z-scan results of S-C_3_N_4_ at (d) 1.06, (e) 1.34, and (f) 1.87 μm, respectively.

As depicted in Figure 3a–c, all the normalized transmittances increased gradually with S-C_3_N_4_ sample approaching to the focus point (*Z* = 0) symmetrically, manifesting its eminent saturable absorption properties. Notably, no nonlinear absorption phenomenon was presented from the utilized quartz substrates even under high power irradiation, proving the credibility of the above experimental results. To quantitatively determine the nonlinear absorption capacity of the S-C_3_N_4_ sample, the effective nonlinear absorption coefficient was employed and it could be extracted by the following equations [42]:

(1) $$T=\sum_{m=0}^{\infty }\frac{{\left[-q_{0}\left(z,0\right)\right]}^{m}}{{\left(m+1\right)}^{1.5}},m\in N\;{\text{q}}_{0}\left(z,0\right)=\frac{\beta_{\text{eff}}L_{\text{eff}}I_{0}}{\left(1+Z^{2}/\left(Z_{0}^{2}\right)\right)}$$

where $L_{\text{eff}}=\left(1-e^{L\alpha_{0}}\right)/\alpha_{0}$ represented the effective thickness, *L* was the actual thickness of the S-C_3_N_4_ nanomaterials, *I*_0_ was the on-axis incident pump intensity and *Z*_0_ was the Rayleigh range. Besides, *α*_0_ and *β*_eff_ was the linear absorption coefficient and nonlinear absorption coefficient, respectively, constituting the absorption model: $\alpha \left(I\right)=\alpha_{0}+\beta_{\text{eff}}I$ . The maximum nonlinear absorption coefficients fitted from the intensity-dependent nonlinear saturation absorption curves were −0.71, −0.82, and −0.66 cm/GW, at 1064, 1342 and 1878 nm, respectively. Compared with the pristine g-C_3_N_4_ (−0.06 cm/GW) [43], the sulfur-doped modified S-C_3_N_4_ featured much larger nonlinear absorption coefficient at 1064 nm, which could be assigned to the optical transition and band filling of the intermediate trap or defect states introduced by sulfur-doping. Moreover, the intensity-dependent modulation depth (Δ*T*) was fitted by the experimental data. It is found that the modulation depth of the modified S-C_3_N_4_ was proportional to the irradiation intensity and the maximum modulation depth was 17.2% at 1.3 μm.

### 3.2 Third-order nonlinear refractive index and susceptibility

In order to determine the effect of sulfur doping in g-C_3_N_4_ on its nonlinear refractive index, we next performed CA Z-scan experiments (more details in Supplementary Material). Under high-intensity laser excitation, nanomaterials showed self-focusing or defocusing effects, resulting in different intensity transmittance through the aperture placed before the detector. Accompanied by the saturable absorption effect in the whole process, therefore, the nonlinear refractive index could be extracted by dividing the CA data by the OA results. The normalized transmittance curves of the CA Z-scan results baselined to the corresponding OA Z-scan data at 1064, 1340, and 1878 nm were depicted in Figure 3d–f. The valley-peak configurations demonstrated the positive nonlinear refractive index, that was self-focusing effects, and its specific values were fitted by the following equation [44]:

(2) $$T_{C/A}=1-\frac{4\Delta \phi \left(z/z_{0}\right)}{\left[1+{\left(z/z_{0}\right)}^{2}\right]\left[9+{\left(z/z_{0}\right)}^{2}\right]}$$

where $\Delta \phi ≅n_{2}I_{0}2\pi L_{\text{eff}}/\lambda$ was on behalf of the phase change and *n*_2_ was the nonlinear refractive index. By fitting the experimental data, the nonlinear refractive indices were calculated to be 9.5, 6.8, and 2.5 × 10^−14^ cm^2^/W at 1.06, 1.34, and 1.87 μm, respectively. In order to eliminate the influence of nonlinear scattering on the determined results, a detector was placed at a certain angle to the direct laser propagation direction, and no nonlinear scattering phenomenon was observed. In fact, thermally induced nonlinear scattering of nanomaterials generally occurs in a solvent [45]. In addition, the effects of thermal load on nonlinearity were more significant for strong radiation intensity. In our case, the relatively low pump intensity was utilized here, the thermo-induced nonlinearity can be ruled out. The presented nonlinear optical properties in the CA Z-scan experiments can therefore be ascribed to S-C_3_N_4_ nanosheets itself.

As shown in Table 1, compared with the nonlinear refractive index of pristine g-C_3_N_4_ [43], after sulfur doping, the *n*_2_ of the sample increased by two orders of magnitude, evidencing again that S doping could indeed enhance the nonlinear optical responses of g-C_3_N_4_ in NIR waveband. What’s more, the third-order nonlinear optical susceptibility, *χ* ^(3)^ was an important parameter to understand the interactions between light and matter in depth. The third-order NLO susceptibility was determined by the real and imaginary parts of *χ* ^(3)^ and these parameters could be derived by the following equations [46]:

(3) $$\text{Re}\left|\chi^{\left(3\right)}\right|=\left[\frac{4\epsilon_{\text{0}}cn_{0}^{2}n_{2}}{3}\right],\text{ Im}\left|\chi^{\left(3\right)}\right|=\left[\frac{10^{-7}cn_{0}^{2}n_{2}\lambda }{96\pi^{2}}\right]\beta_{\text{eff}},\;\left|\chi^{\left(3\right)}\right|=\sqrt{{\left|\text{Im}\left|\chi^{\left(3\right)}\right|\right|}^{2}+{\left|\text{Re}\left|\chi^{\left(3\right)}\right|\right|}^{2}}$$

where *c* was the speed of light in vacuum, *ε*_0_ reflected vacuum dielectric constant and *n*_0_ was the linear refractive index of the sample. All the fitted nonlinear parameters of the S-C_3_N_4_ sample at different wavelengths were listed in Table 1. Additionally, the comparison of the nonlinear characteristics between the S-C_3_N_4_ sample and some current mainstream two-dimensional materials was summarized in Table 1 as well. By comparison and analysis, the modified S-C_3_N_4_ sample has a larger nonlinear refractive index and third-order NLO susceptibility than other thin-film materials, indicating the remarkable potential as a modified NLO nanomaterial for optical application.

**Table 1:** The broadband nonlinear parameters of S-C_3_N_4_ and its comparison with that of other two-dimensional materials.

Materials	*λ* (nm)	*β*_eff_ cm/GW	Δ*T* (%)	*n*_2_ (cm^2^/W)	Re|*χ* ^(3)^| (*esu*)	Im|*χ* ^(3)^| (esu)	|*χ* ^(3)^| (*esu*)	Refs.
**S-C**_**3**_ **N**_**4**_	1064	−0.71	11.3	9.5 × 10^−14^	1.51 × 10^−11^	9.53 × 10^−13^	1.51 × 10^−11^	This work
	1342	−0.82	17.2	6.8 × 10^−14^	1.08 × 10^−11^	1.39 × 10^−12^	1.09 × 10^−11^	
	1878	−0.66	8.0	2.5 × 10^−14^	3.96 × 10^−12^	1.56 × 10^−12^	4.26 × 10^−12^	
**g-C**_**3**_ **N**_**4**_	1064	−0.06	─	3.4 × 10^−16^	2.3 × 10^−13^	2.20 × 10^−13^	2.21 × 10^−13^	[43]
**MXene**	1064	−0.206	38.3	3.47 × 10^−16^	3.53 × 10^−14^	∼10^−13^	∼10^−13^	[47]
	1800	−0.112	28	9.86 × 10^−16^	1.01 × 10^−13^	∼10^−13^	∼10^−13^	
**MoSe**_**2**_	1064	─	10.5	2 × 10^−13^	8.2 × 10^−12^	1.61 × 10^−12^	8.36 × 10^−12^	[48]
**WS**_**2**_	1064	−5.1	─	5.83 × 10^−11^	2.31 × 10^−8^	1.75 × 10^−11^	2.3 × 10^−8^	[49]

### 3.3 Broadband S-C_3_N_4_ based PQS lasers application

**Figure 4:**
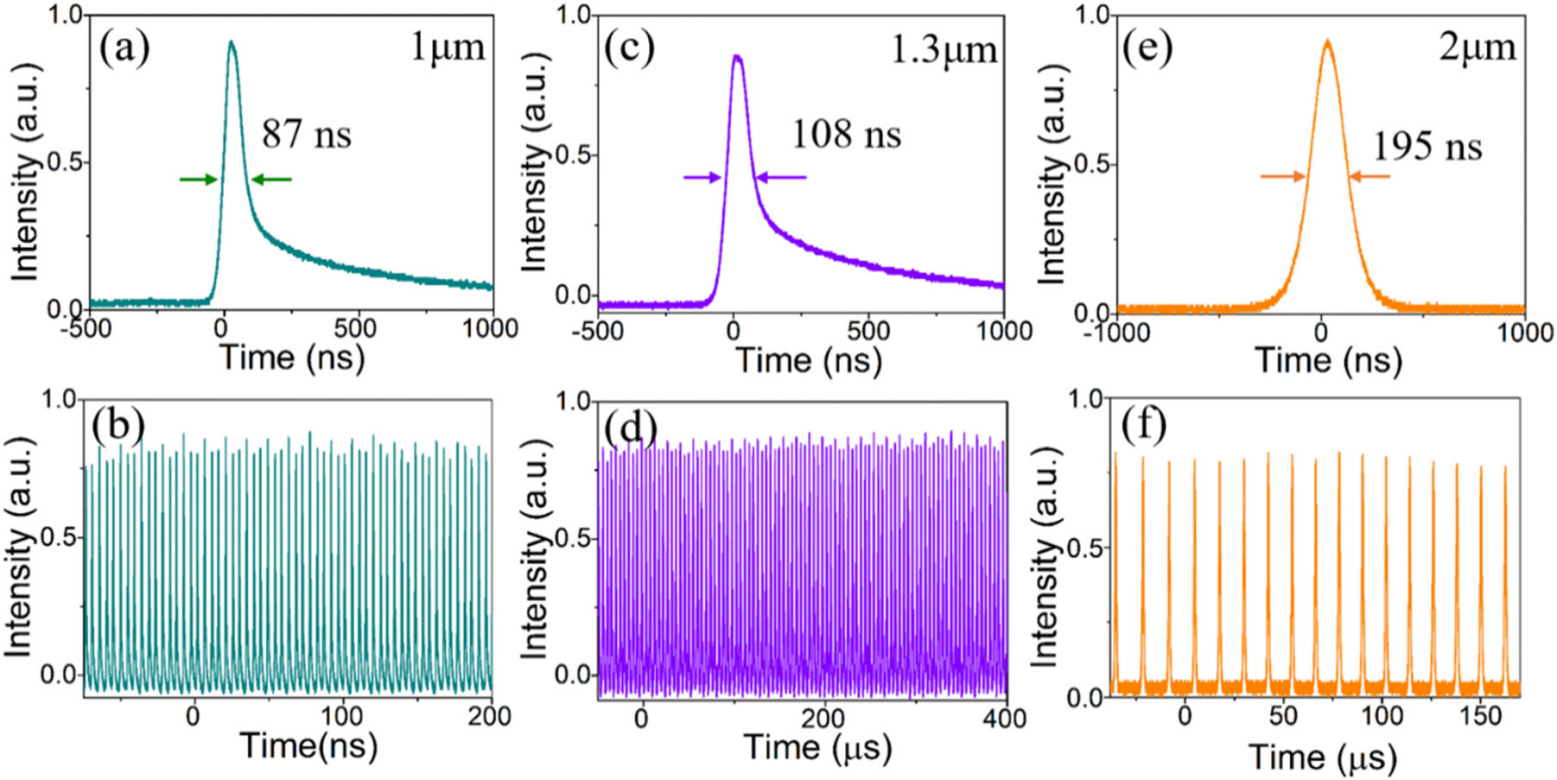
Experimental performances of the PQS lasers with CuO and TiO_2_/CuO nanocomposites at 1.06 μm. (a) CW and Q-switched average output power. (b) Pulse width and repetition rate versus the incident pump power. (c), (d) Typical Q-switched temporal pulse profile and trains of CuO and TiO_2_/CuO lasers, respectively.

The above experiments illustrated that sulfur doping had an apparent enhancement on the nonlinear absorption characteristics of g-C_3_N_4_, motivating us to further apply the modified S-C_3_N_4_ to broadband PQS lasers as an optical modulator. The employed laser resonators were compact two-mirror straight cavities, and the more experimental details were illustrated in Supporting Information. The average output power of the continuous-wave (CW) and Q-switching lasers as the functions of the incident pump power at 1064, 1342 and 1878 nm were shown in Figures S3–5, respectively. Founding that the output power practically increased linearly with the pump power and the maximum slop efficiency was 21.3% at 1.06 μm. It’s worth noting that no thermal damage was observed on the surface of S-C_3_N_4_, and the output power remained almost unchanged even with adjusting the sample laterally during laser operations, demonstrating the great uniformity, high thermochemistry and modulation stability. Furthermore, the pulse compression capability was an important indicator to judge the quality of a SA, so the pulse width versus the incident pump power was depicted in Figures S3b–5b. The obtained minimum pulse duration was measured to be 87, 108, and 195 ns at 1064, 1342, and 1878 nm, corresponding to the repetition rate of 232, 214, and 84 kHz, respectively. The pulse width within hundred nanoseconds manifested plenty the remarkable pulse compression performance of the modified S-C_3_N_4_ sample. What’s more, the transient snapshots of single pulse profiles and pulse sequences were recorded in Figure 4, and the root mean square error (RMSE) of the pulse-to-pulse amplitude instabilities at different wavelengths were calculated to be less than 2%, evidencing the high modulation stability of S-C_3_N_4_.

The calculated single pulse energy and peak power versus the incident peak power were presented in Figures S3–5c. The single pulse energy gradually tended to be saturated with increasing the pump power, and the maximum peak was 10.1, 8.5, and 13.7 W, at 1064, 1342, and 1878 nm, respectively. Besides, it’s worth emphasizing that the S-C_3_N_4_ nanomaterials were uniformly dispersed on the substrates. During synthesis, the prepared homogeneous supernatants were spin-coated on the substrates for several times and only a small amount was taken each time. What’s more, the PQS laser pulse outputs obtained by moving the S-C_3_N_4_ sample transversely were almost constant, which demonstrated the high uniformity of the S-C_3_N_4_ on the substrates. To comprehensively analyze the laser performances of the S-C_3_N_4_ sample, we compared it with different SAs on the output characteristics. As summarized in Table 2, the S-C_3_N_4_ featured extraordinary pulse compression capability and great potential to generate high peak power, providing a noteworthy reference for the development of laser devices based on nanomaterials.

**Table 2:** The laser output performance comparison of S-C_3_N_4_ with different SAs at 1, 1.3, and 2 μm.

Material	*λ* (nm)	Pulse width (ns)	Rate (kHz)	Output power (mW)	Peak power (W)	Refs.
**g-C**_**3**_ **N**_**4**_	1320	275	154	960	─	[43]
	1942	390	65	331	4.76	[50]
**MoS**_**2**_	1079	227	232	260	4.9	[51]
	1342	188	73	144	9	[52]
	1902	120	48	100	2.6	[53]
**BP**	1064	495	312	22	0.14	[54]
**S-C**_**3**_ **N**_**4**_	1064	87	232	204	10.1	This work
	1342	108	214	197	8.5	
	1878	195	84	225	13.7	

### 3.4 Broadband ultrafast photonics applications

**Figure 5:**
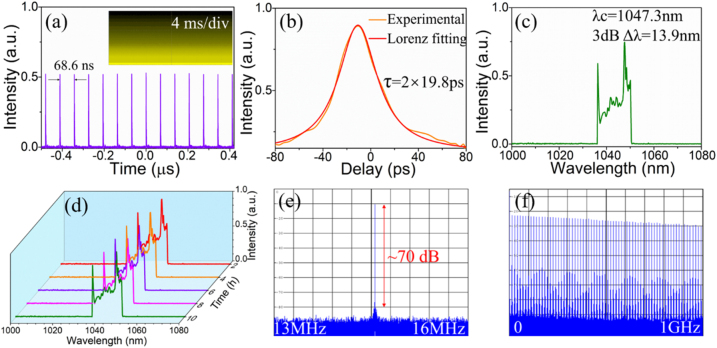
Output characteristic of the S-C_3_N_4_ based mode-locked YDFL. (a) Pulse train with different scale. (b) Autocorrelation trace. (c) Emission spectrum. (d) Evolution of the spectrum in 10 h. (e) RF fundamental frequency spectrum. (f) RF spectrum with 1 GHz span.

To further investigate the ultrafast response property of the modified S-C_3_N_4_ sample, it was inserted into all-fiber ring cavities to explore the mode-locking laser performances. The employed experimental schematics were shown in Figure S5. For the 1 μm YDFL, the threshold pump power of the mode-locking operation was 200 mW. Under a maximum pump power of 500 mW, the highest output power was 4.86 mW. Figure 5 demonstrated the obtained high stable mode-locking performances at the pump power of 500 mW. As illustrated in Figure 5a, the pulse period was 68 ns, corresponding to a repetition rate of 14.58 MHz, which agreed well with the cavity length. The longer pulse train was shown in the inset of Figure 5a with a span of 40 ms, evidencing the high stability of the mode-locking operation. Notably, without S-C_3_N_4_ in the cavity, no mode-locking operation was observed no matter how the PC and pump power were adjusted, indicating the dominance of the sample for the starting of mode-locking. The autocorrelation trajectory was shown in Figure 5b. Assuming Lorenz pulse profile, the pulse duration was 19.8 ps. Then, a snapshot of its corresponding emission spectrum was taken, as illustrated in Figure 5c. The spectrum presented an obvious gate-shaped spectrum with steep edges on both sides, indicating a typical dissipative soliton mode-locking state. The center wavelength of the soliton pulse was located at 1047.3 nm and the 3 dB bandwidth was about 13.9 nm. To investigate the stability of the mode-locked spectrum, we monitored it for 10 h. As depicted in Figure 5d, the spectral shapes and locations were constant, manifesting the eminent stability. Accordingly, the time-bandwidth product (TBP) of the mode-locked YDFL was calculated to be 75.2. The large deviation with the transmission limit of 0.142 demonstrated that the pulses were highly chirped, similar to most Yb-doped dissipative soliton mode-locked lasers [55], [56], [57]. However, a shorter pulse duration could be achieved by compensating for the cavity dispersion. As illustrated in Figure 5e, the RF spectrum exhibited a high signal-to-noise ratio (SNR) of around 70 dB and its location matched well with the repetition rate, revealing the fundamental frequency mode-locking operation. Figure 5f recorded the corresponding RF with 1 GHz span, which again confirmed the stability of the constructed YDFL.

**Figure 6:**
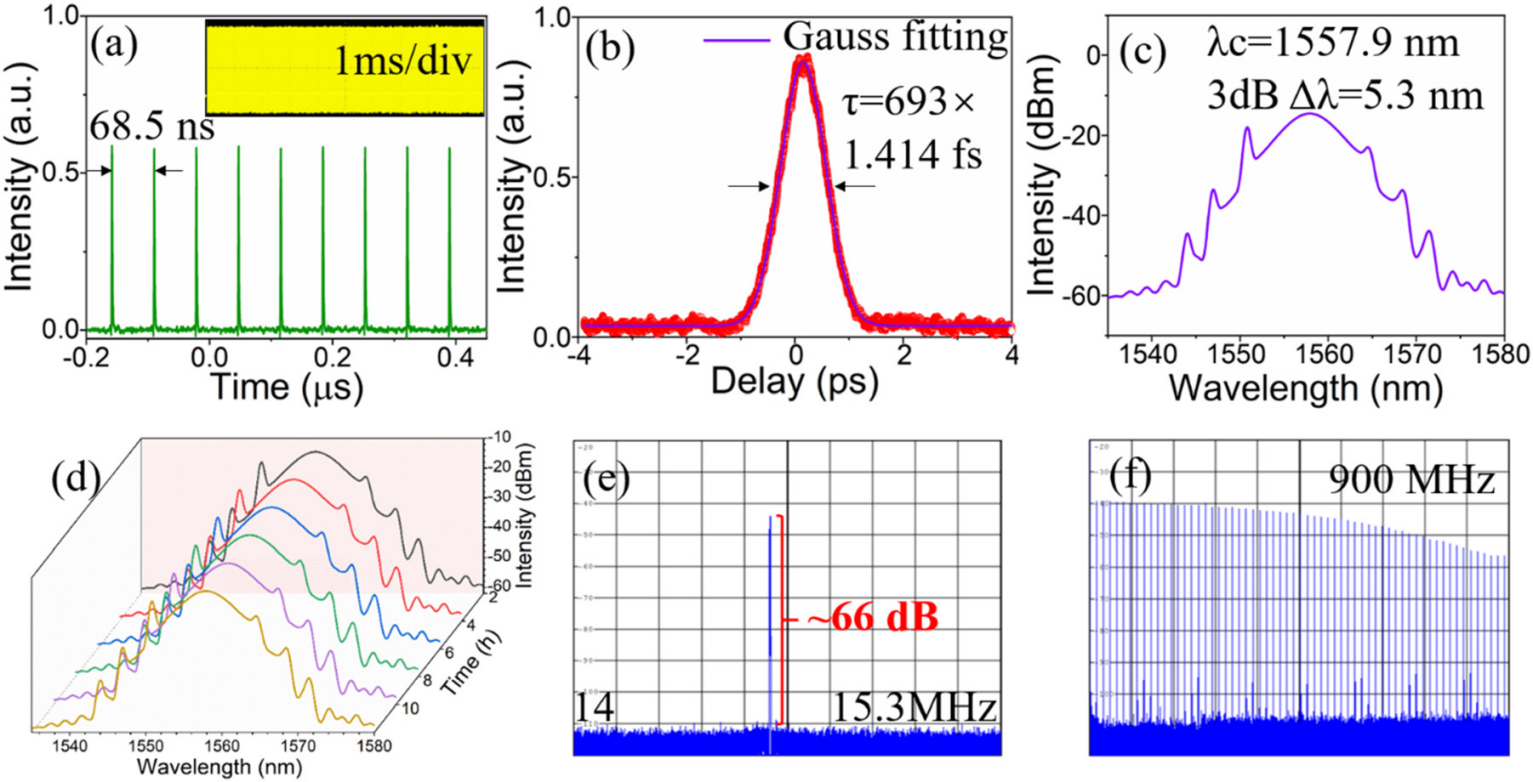
Output characteristic of the S-C_3_N_4_ based mode-locked EDFL. (a) Pulse train with different scale. (b) Autocorrelation trace. (c) Emission spectrum. (d) Evolution of the spectrum in 12 h. (e) RF fundamental frequency spectrum. (f) RF spectrum within 900 MHz.

When it was inserted into the erbium-doped fiber (EDF) laser cavity as a saturable absorber, setting the pump power to be 120 mW and adjusting the PC, stable mode-locking operation was realized at 1550 nm. Figure 6 recorded the laser output performances at the pump power of 350 mW. As depicted in Figure 6a, the transient pulse sequence with 68.5 ns interval between two adjacent pulses was presented. Assuming a Gaussian profile, the pulse width was fitted to be 693 fs. The output spectrum with the center at 1557.9 nm was attached with distinct Kelly sidebands, characterizing the traditional soliton mode-locking state. The TBP was calculated to be 0.454, close to the theoretical limit of sech^2^ model (0.441). This revealed that the cavity contained a weak chirp, which was in line with the characteristics of traditional soliton mode-locking pulse. What’s more, the long-time operation stability of the mode-locking operation is an important parameter. There was almost no deformity and shift in the spectrum evolution for up to 12 h, as shown in Figure 6d, which illustrated the excellent modulation stability of S-C_3_N_4_ nanosheets. The measured RF spectrum with a fundamental frequency of 14.60 MHz corresponding well with the above-mentioned round-trip time, possessed a high SNR of around 66 dB. As depicted in Figure 6e, even within the broader 900 MHz bandwidth, no extra noise frequency disturbed the laser operation, well demonstrating the high stability of the EDF mode-locking laser. In addition, the constructed YDF and EDF lasers were kept operating uninterruptedly for more than 72 h, and the lasers could still maintain good mode-locking operation. Therefore, all these definitely demonstrated that the S-C_3_N_4_-based NIR ultrafast lasers were highly stable in long-term operations. In order to have a comprehensive acknowledgement of the S-C_3_N_4_ nanosheets’ laser performances, we compared it with some other mainstream 2D nanomaterials both in YDF and EDF lasers. As summarized in Table 3, the S-C_3_N_4_ based YDFL featured a much shorter pulse duration and large potential in ultrafast laser generation in the near-infrared bands.

**Table 3:** Performance comparisons of the S-C_3_N_4_-based YDF and EDF lasers with some mainstream 2D nanomaterials.

SA	Wavelength (nm)	Pulse width (ps)	Repetition rae (MHz)	Output power (mW)	Refs.
**Graphene**	1064.1	2300	1.072	0.19	[55]
**MoS**_**2**_	1054.3	800	6.55	9.3	[56]
**WS**_**2**_	1063.6	630	5.57	7.6	[57]
	1563.8	0.808	19.57	2.64	[58]
**Ti**_**3**_ **C**_**2**_ **T**_***x***_	1065.89	480	18.96	9	[47]
**ReS**_**2**_	1563	1.247	3.438	60	[59]
**S-C**_**3**_ **N**_**4**_	1047.3	19.8	14.58	4.86	This work
	1557.9	0.693	14.60	2.12	

## 4 Theoretical calculation and discussion

**Figure 7:**
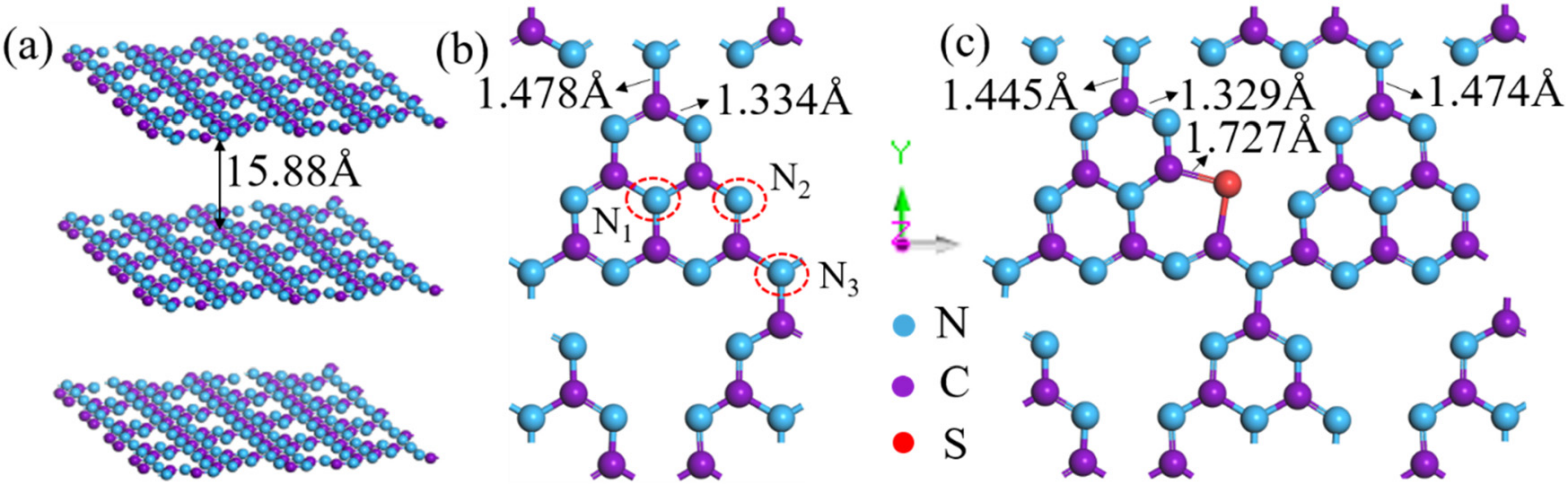
(a) The perspective view, (b) top view of the geometric model of pure g-C_3_N_4_. (c) The top view of S-C_3_N_4_.

To deeply investigate the intrinsic mechanism of sulfur doping on the broadband NIR optical response of g-C_3_N_4_, the structure and band gap variation was simulated by first-principle calculation, as implemented in VASP. Employing the generalized gradient approximation (GGA) with Perdews–Burkes–Ernzerhof (PBE) functional for geometric optimization and single-point energy calculations [60], [61], [62]. The Heyd–Scuseria–Ernzerhof (HSE06) hybrid functional correction method was employed to determine and validate the electronic and energy band properties [63]. The Monkhorst–Pack k-points and cutoff energy were tested to be 2 × 2 × 1 and 500 eV, respectively [64]. Good convergences were ensured by optimizing all atomic positions till the total energy convergence was less than 1.0 × 10^−6^ eV and atomic forces allowance was less than 1.0 × 10^−2^ eV/Å. The initial heptazine-based monolayer g-C_3_N_4_ structure was modeled. The opted geometric structure was shown in Figure 7a–b, with an interlayer spacing of 15.88 Å. In order to ensure the validity of the S doping model in theoretical calculations, we selected three different positions of N atoms in the g-C_3_N_4_ structure based on symmetry and labeled them as N_1_, N_2_, and N_3_, as shown in Figure 7b. Subsequently, a single S atom was utilized to replace these three positions in a 2 × 1 supercell. The calculation results showed that the S-doping system at the N_2_ position featured the lowest total energy, which indicated that in actual situations, this structure was the most stable being and appearing. Therefore, our next discussion was all based on the S atom substitution model at the N_2_ position.

**Figure 8:**
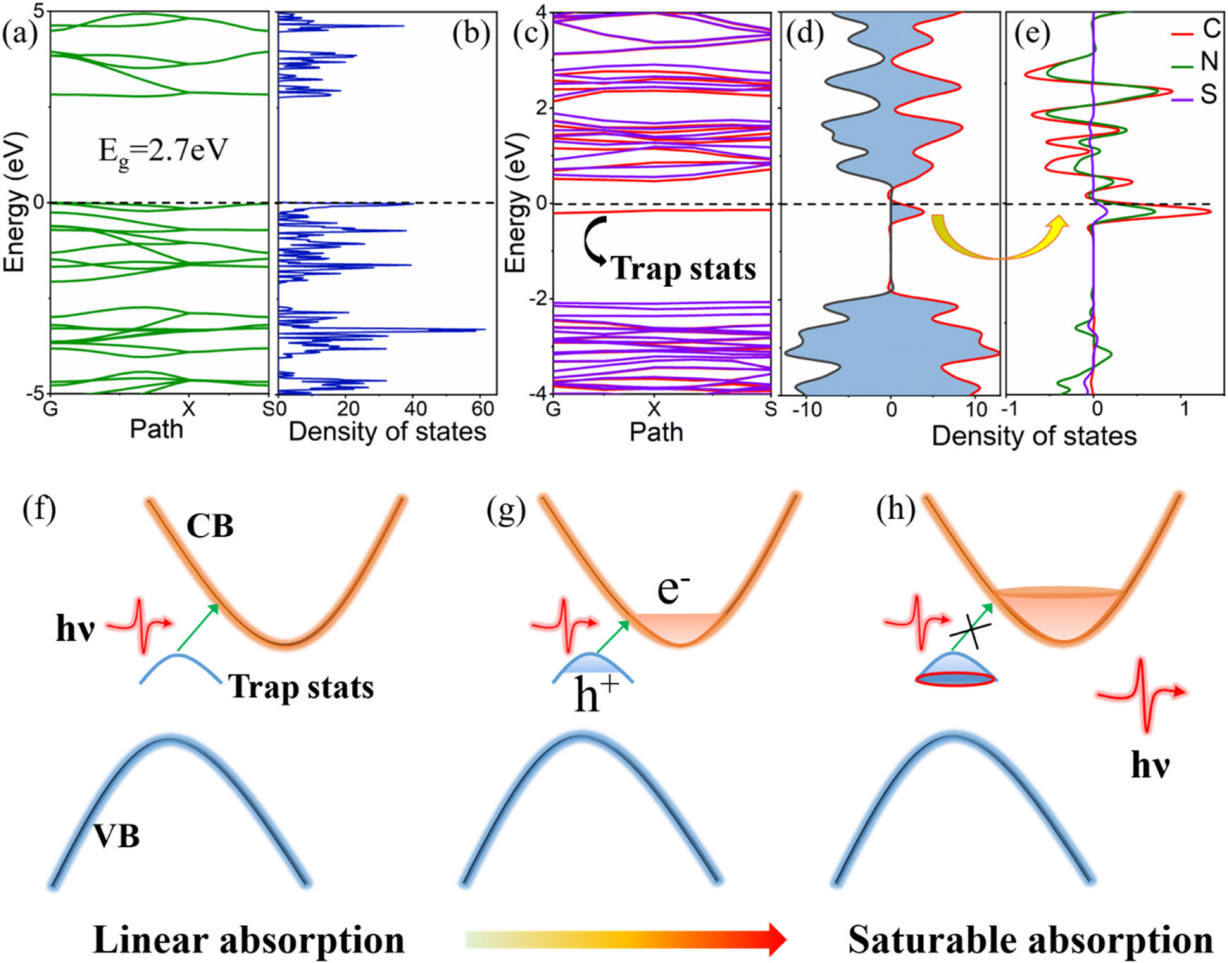
(a) The bandstructure, (b) total density of states of pure g-C_3_N_4_. (c) The bandstructure, (d) total density of states, (e) partial density of states of S-C_3_N_4_. (f–h) The schematic diagram of the saturable absorption mechanism of S-C_3_N_4_.

To obtain the stable structure of sulfur-doped g-C_3_N_4_, geometric optimization was performed on it. As shown in Figure 7c, no obvious deformation of geometric structure was observed after S-doping, and only a small distortion occurred near the S atom. To further illustrate the structural change after doping, we measured the C–N bond length, as labeled in Figure 7c. The C–N bond length near the S atom was significantly decreased while others only slightly changed. This might be due to the difference in radius and electronegativity of the impurity S atom from the N, which caused a certain degree of deformation after doping. In addition, the length of the formed C–S bond was 1.727 Å. So as to qualitatively grasp the influence of S doping on the electronic structure characteristics of g-C_3_N_4_, we have carried out theoretical calculations and analyses on the energy band and the density of states (DOS). Firstly, as shown in Figure 8a and b, the pure band gap (*E*_*g*_) of the heptazine-based g-C_3_N_4_ was 2.7 eV, consisting well with Ref. [65]. The DOS calculation result in Figure 8b further confirmed it, and no magnetic was found. Figure 8c–e showed the theoretical simulation results of the energy band and DOS of S-doped g-C_3_N_4_. The incorporation of sulfur atoms into g-C_3_N_4_ matrix had a significant effect on the band structure and electronic properties of g-C_3_N_4_. Obviously, an impurity level appeared below the Fermi level in the forbidden band, showing that the disturbance of the crystal lattice caused by sulfur doping resulted in energy level splitting of the coordinated C atoms. More finely, the upward and downward spin DOS near the Fermi level were asymmetrical, which indicated that anisotropic spin splitting phenomenon occurred in g-C_3_N_4_. It could be ascribed to that the introduced excess electrons into the system after sulfur doping distorted the magnetic field distribution, causing the spin splitting of carbon and nitrogen around the sulfur dopant. Therefore, the S-C_3_N_4_ system was magnetic and the net magnetic moment of the electron spin obtained was about 1 *μ*_*B*_. The electrons on the impurity energy level would transfer to the conduction band under light excitation, which would be conducive to the red shift of the absorption spectrum and increase the absorption response of carbon nitride in the near-infrared band. Therefore, the internal physical mechanism of the abovementioned nonlinear saturable absorption of S-C_3_N_4_ in broadband NIR wavebands could be interpreted by the constructed model, as shown in Figure 8f–h. The defects level, as trap states, was one of the absorption and recombination center. When the photon density in the cavity was too low to cause nonlinear optical properties of the S-C_3_N_4_, it showed linear transmission and the transmittance was small signal transmittance. As the photon density in the cavity further increased, massive electrons in the trap states were excited to the conduction band until all electrons in the trap state were evacuated. At this time, the photons were no longer being absorbed, and the sample presented a bleached and transparent state, and the photons would pass through the S-C_3_N_4_ without any loss. It was this nonlinear transmission mechanism that endowed the material with the saturable absorption properties in NIR region. In addition, this trap state could also be used as a recombination center to trap electrons and shorten the lifetime of unbalanced carriers [66], which was beneficial for ultrafast optical response and ultrafast photonics applications.

## 5 Conclusions

In summary, the S-C_3_N_4_ nanomaterials were fabricated by hydrothermal method and liquid-phase exfoliation method. A series of morphology and structure characterizations demonstrated the massive porous surface morphology of S-C_3_N_4_ and effectiveness of sulfur doping. Then the as-synthesized S-C_3_N_4_ nanomaterials with eminent broadband nonlinear optical performances for near-infrared application were demonstrated via sulfur doping of g-C_3_N_4_. The modified g-C_3_N_4_ delivered large effective nonlinear absorption coefficients of −0.71, −0.82, and −0.66 cm/GW at 1.06, 1.34, and 1.87 μm, respectively. The enhanced nonlinear absorption properties of g-C_3_N_4_ in NIR bands could be attributed to the spin-splitting and defect level caused by sulfur doping. Besides, the extraordinary optical modulation performances, including short pulse duration of 87 ns, large peak power and high modulation stability, manifested the superb capability of the modified S-C_3_N_4_ as a passive modulator. Furthermore, the broadband ultrafast mode-locking operation had been verified in YDF and EDF lasers, generating a highly stable dissipative soliton with a pulse duration of 19.8 ps and a ultrashort traditional soliton with a pulse width of 693 fs, respectively. What’s more, the density functional theory calculation demonstrated that sulfur-doping introduced defects level and caused anisotropic spin splitting in g-C_3_N_4_ beneficial to the nonlinear optical absorption characteristics of S-C_3_N_4_ in NIR regime. The presented sulfur-doped g-C_3_N_4_ nanomaterials with eminently outstanding nonlinear optical performances might explicitly promote the development and application of g-C_3_N_4_ in advanced NIR optoelectronic and ultrafast photonic devices.

## Supplementary Material

Supplementary Material
