# Supplementary material for: Band structure tuning of g-C3N4 via sulfur doping for broadband near-infrared ultrafast photonic applications
Source: Nanophotonics. 2021 Nov 17;11(1):139–51. doi: 10.1515/nanoph-2021-0549 (PMC11502033; doi:10.1515/nanoph-2021-0549)
Supplement: Supplementary file 1 — Supplementary Material [file j_nanoph-2021-0549_suppl_001.docx]

**Supporting information of “Band structure tuning of g-C_3_N_4_ via sulfur doping for broadband near-infrared ultrafast photonic applications”**

*Li Dong^1^, Hongwei Chu^1^*, Shiping Xu ^2^, Ying Li^3^, Shengzhi Zhao^1^, and* *Dechun Li^1^**

^1^School of Information Science and Engineering, Shandong University, Qingdao 266237, China

^2^ School of Environmental Science and Engineering, Shandong University, Qingdao 266237, China

^3^Key laboratory of Colloid and Interface Chemistry, Minister of Education, and School of Chemistry and Chemical Engineering, Jinan 250100, China

Z-scan experiments

The open-aperture (OA) Z-scan experimental setup was depicted in Figure S1. The pulse width and repetition rate of the pump sources at 1.06, 1.34, and 1.87 μm were 50 ns and 3 kHz. A 1:1 beam splitter was used to divide the pump laser into two beams, one as the reference laser and the other was the detection laser. Due to the focusing effect of the lenses, the peak power densities irradiated on the sample were different at different locations of the z-axis. The sample was moved along the z-axis via a motion controller (Zolix. SC300-1B). The focal positions of the lenses were calibrated by employing a uniform CVD grown GaAs nanosheets. The transmitted power was monitored by a dual-channel energy meter (Thorlabs. PM320E). Nonlinear absorption properties were determined by measuring the transmittances at different positions. The power was recorded using a PM320E (Thorlabs Inc.) dual-channel energy meter. What’s more, for the close-aperture (CA) experiments, a small aperture was put immediately after the sample to limit the transmitted beam irradiating the detector. By measuring the relationship between the beam transmittance through the aperture and the distance z from the focal point of the sample, the nonlinear refractive index of the sample could be calculated.


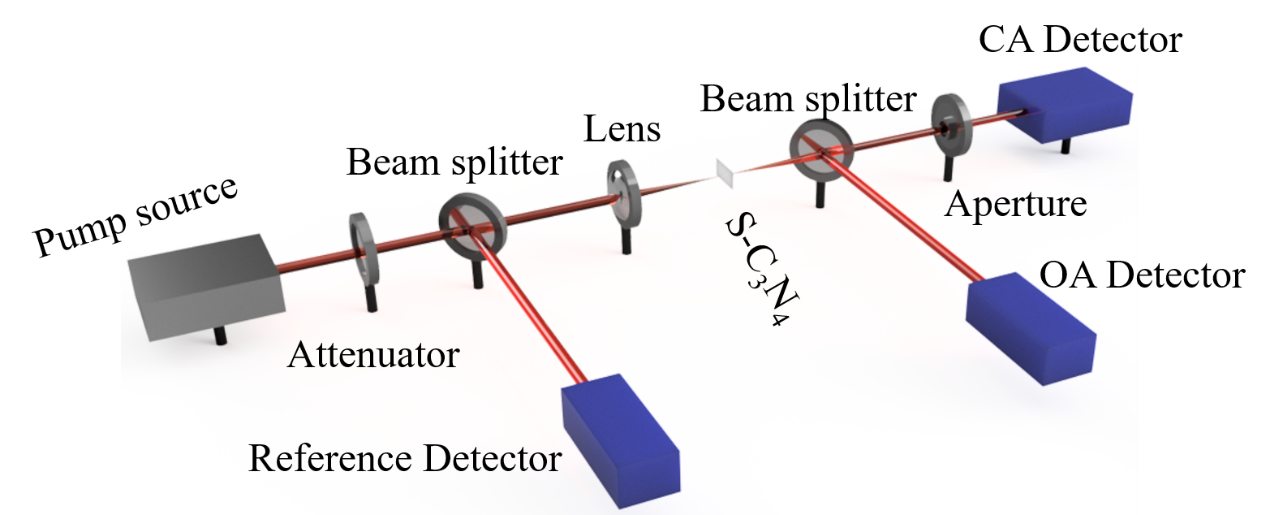


**Figure S1:** Schematic of open-aperture Z-scan experiment..

**PQS laser experiments construction:**

For the PQS lasers experiments, the compact two-mirror resonators were utilized, as shown in Figure S2. The fiber-coupled pump source (numerical aperture NA=0.2, fiber core diameter of 400 μm, FAP system, Coherent Inc, USA) was focused through a 1:2 focusing system to the input mirror M1. The M1 was antireflection coated (AR) of the incident pump laser and high reflectivity (HR>99.9%) coated for the laser wavelength. To manage well the accumulated heat inside the crystals, the crystals wrapped with indium foil, were mounted on the copper holders with circulating water at 15 °C. Besides, the S-C_3_N_4_ sample was placed close to the output mirror M2 to reduce the thermal damage on the surface of S-C_3_N_4_. The OC M2 was highly transparent to the pump laser and had different output transmittance proportions of the signal laser. The specific parameters of the mirrors and pump sources were listed in Table S1. Using a filter to block the residual pump power. Moreover, the average output power was detected by a power meter (MAX 500AD, Coherent Inc.). The Q-switched pulse temporal profile was monitored with an InGaAs detector (EOT) and observed by a phosphor oscilloscope (DPO 7104C, Tektronix Inc.).


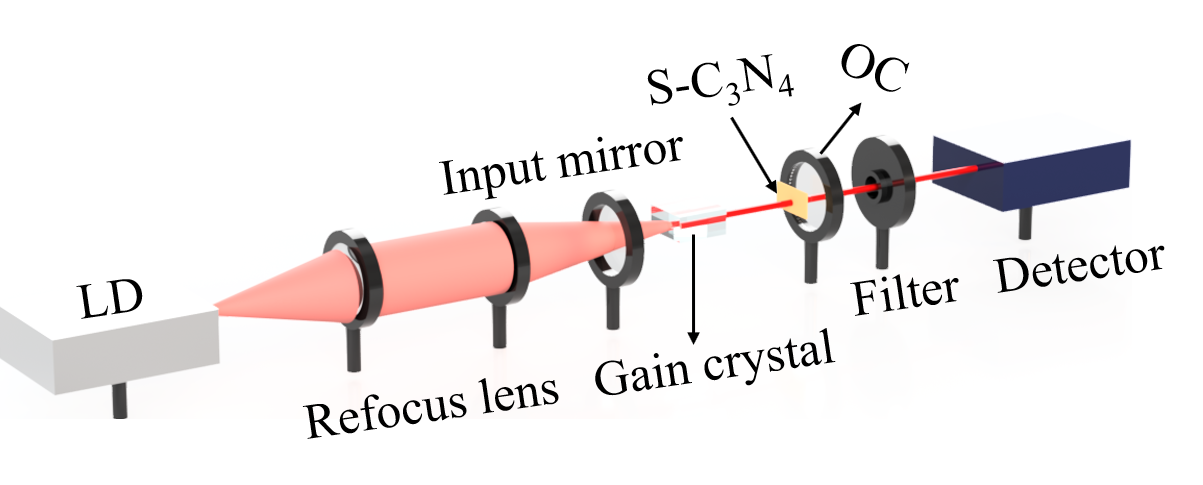


**Figure S2:** Schematic diagram of the PQS lasers.

| **Signal**  **(nm)** | **Pump**  **(nm)** | **Input mirror** | **Gain medium** | **Output mirror** | **Cavity length (mm)** |
| --- | --- | --- | --- | --- | --- |
| 1064 | 808 | Plane mirror  808nm-HT  1064nm-HR | c-cut 0.1 at.%+0.3 at.%+0.8 at.% doped, 3×3×(3+3+4) mm^3^ composite Nd:GdVO_4_ | T=20% | 40 |
| 1342 | 808 | Plane mirror  808nm-HT  1342nm-HR | c-cut 0.1 at.%+0.3 at.%+0.8 at.% doped, 3×3×(3+3+4) mm^3^ composite Nd:GdVO_4_ | T=3.8% | 25 |
| 1878 | 794 | Concave mirror  794nm-HT  1878nm-HR | a-cut 3.0 at.% doped,  3×3×10 mm^3^ Tm:YLF | T=1% | 20 |

Table S2: Specific parameters of the mirrors and pump sources.

**PQS laser output performances**

1. **1064 nm**

**
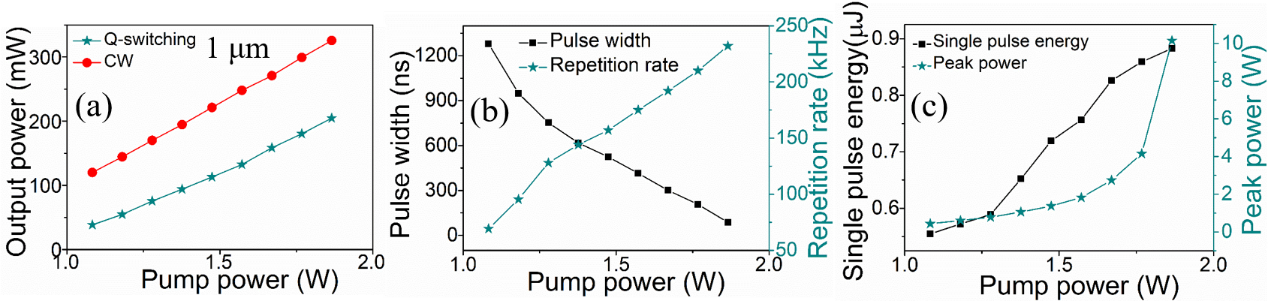
**

**Figure S3.** Laser performance at 1 μm. (a) Output power, (b) Pulse duration and repetition rate, (c) single pulse energy and peak power versus the pump power.

1. **1342 nm**

**
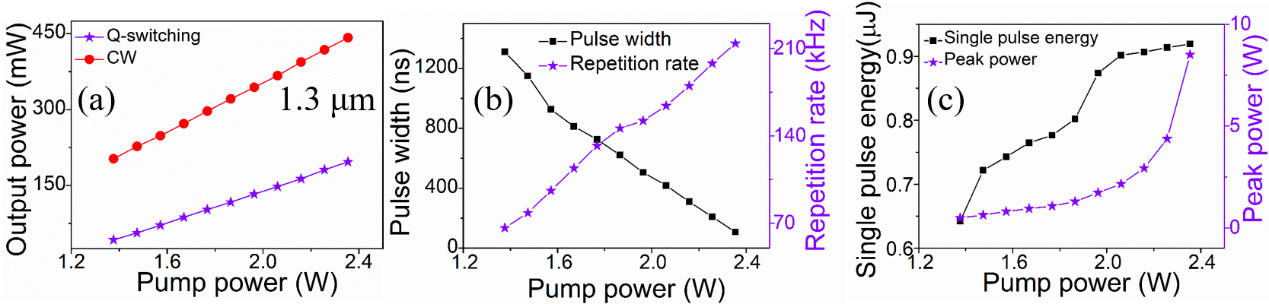
**

**Figure S4.** Laser performance at 1.3 μm. (a) Output power, (b) Pulse duration and repetition rate, (c) single pulse energy and peak power versus the pump power.

1. **1878 nm**

**
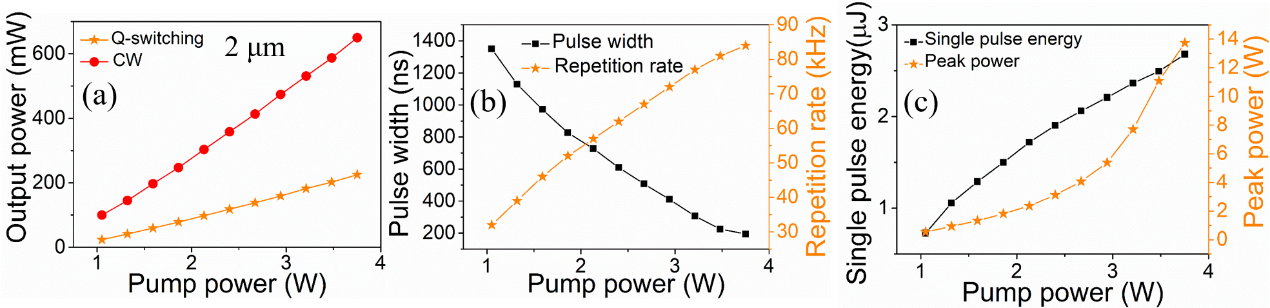
**

**Figure S4**. Laser performance at 2 μm. (a) Output power, (b) Pulse duration and repetition rate, (c) single pulse energy and peak power versus the pump power.

**Ultrafast laser construction:**


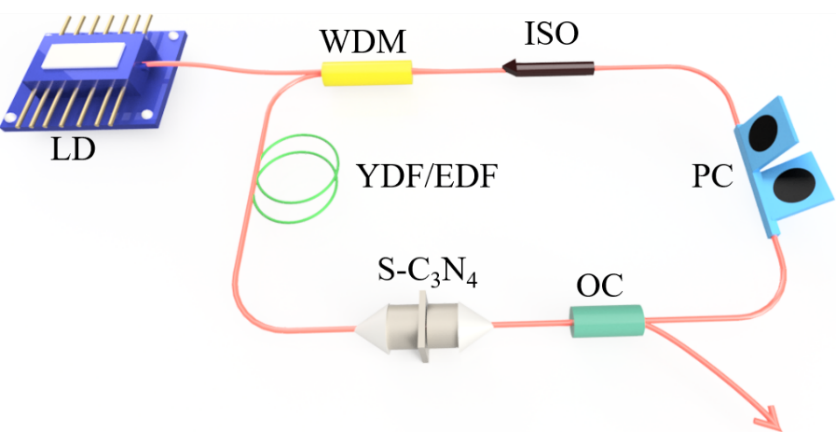


**Figure S5**. Schematic of the S-C_3_N_4_ based YDFL and EDFL resonator.

As shown in **Figure S**5, employing a backward pumping scheme, a 3.5 m-long ytterbium-doped fiber with a peak core absorption of 80 dB/m and a 0.6 m long erbium-doped fiber (Er 110, 4/125) as the gain medium was pumped by a 976 nm laser diode through a 980/1030 nm and 976/1550 nm wavelength division multiplexer (WDM), respectively. A polarization-independent isolator (PIISO) was utilized to force the unidirectional light transmission. A polarization controller (PC) was employed to adjust the polarization states in the cavity. The S-C_3_N_4_ based SA device was prepared by laser deposition method. Firstly, a fiber patch cord was fused to a 976 nm fiber diode pump source. The cleaned FC/APC end face was inserted into the as-prepared S-C_3_N_4_ supernatants for deposition. Then, increase the pump power until the output power from the fiber patch cord was around 20 mW and the deposition lasted for an hour. Finally, take it out and place it in a dry box to fully dry. Upon that, the S-C_3_N_4_ based SA device was successfully fabricated. The insertion loss of the S-C_3_N_4_ device was measured to be around 1.86 dB. The 10 % laser was extracted to monitor the laser performances. The output pulses sequences were recorded by a 1 GHz oscilloscope (Tektronix MDO4104C) with a high-speed PIN photodetector and an optical spectrum analyzer (Anritsu MS9740B) was used to determine the output spectra. Moreover, in order to analyze the spectral components and evaluate the signal-to-noise ratio, a radio frequency (RF) spectrum analyzer was utilized.
